# Tracheal transplantation: is there *lumen* at the end of the tunnel?

**DOI:** 10.1590/S1516-31802009000500001

**Published:** 2010-02-03

**Authors:** Paulo Manoel Pêgo-Fernandes, Artur Eugênio de Azevedo-Pereira

**Affiliations:** I MD, PhD. Associate professor of the Department of Cardiopneumonology, Faculdade de Medicina da Universidade de São Paulo (FMUSP), São Paulo, Brazil.; II MD. General thoracic surgeon and PhD student in the Department of Cardiopneumonology, Faculdade de Medicina da Universidade de São Paulo (FMUSP), São Paulo, Brazil.

The good results achieved from vital organ transplantation around the world have motivated research on non-vital organ transplantation. Recent reports on arm, ovary and airway transplantation have been published.^1^ On the other hand, tracheal transplantation is not a new idea. The simple anatomical structure of the trachea has misled researchers since the first attempts at tracheal transplantation in the late 19^th^ and early 20^th^ century.^2^ Since then, a large number of surgical approaches for tracheal transplantation have been tried, with poor results.^2^ In spite of some anecdotal reports, no clinically useful standard approach for tracheal transplantation has yet been achieved, and this remains under investigation.

The development of a clinically useful approach relies on both a technically simpler way for tracheal graft vascularization, and the avoidance of post-transplantation immunosuppressive therapy. Recently, tracheal graft vascularization with topically-applied angiogenic factors has been reported.^3^ The use of angiogenic factors could obviate the need for complex surgical procedures for tracheal vascularization. Moreover, the recently described reepithelialization phenomena, and reports on improvements through tissue-engineering techniques, have raised the possibility of abbreviated or even absence of post-transplantation immunosuppressive therapy.^4^^,^^5^

What kind of patients would benefit from tracheal transplantation? The indications for tracheal transplantation would be long-length tracheal lesions for which tracheal resection with end-to-end anastomosis (tracheoplasty) was impossible.^2^ Long-length tracheal stenosis or malacia, chiefly secondary to airway intubation, would be the most common indication. Other indications would be tumors involving a large extent of the trachea, and relapsing tracheal stenosis occurring after a previous attempt at surgical treatment. Currently, these patients are palliated with airway prostheses. Silastic T tubes are used in virtually all these cases. However, these tubes need to be changed on a regular basis, and complications with these devices occur in more than one third of such patients.^6^

How many patients would benefit from tracheal transplantation in Brazil? According to data from the Brazilian National Health System (Sistema Único de Saúde, SUS), 1,688 airway interventions were performed in 2008.^7^ These interventions included 248 cases of airway stenting, 849 of tracheoplasty and 572 of laryngotracheoplasty. Among patients who undergo airway stenting, around 70% are thought to have long-length benign stenosis that is not manageable with tracheoplasty. In addition, about 5% of patients undergoing tracheoplasty are expected to develop postoperative stenosis.^2^ Therefore, from a rough estimate, about 200 patients per year could benefit from tracheal transplantation in Brazil. In spite of being a small group, this pool of patients could achieve a definitive solution through tracheal transplantation.

However, besides the technical challenges, there are also ethical concerns. In order to be tested within a clinical scenario, tracheal transplantation needs to be at least as safe as airway prosthesis. Indeed, some researchers question the worth of expensive and technically demanding procedures for non-vital organ transplantation.^1^ Although recent advances have renewed the interest in tracheal transplantation among investigators, there are still some questions to be resolved before tracheal transplantation could be clinically useful. Nonetheless, we are sure that tracheal transplantation will become an important procedure for dealing with complex airway lesions in the near future.
